# Global trends and career development of human resources for paediatrics: a systematic review

**DOI:** 10.7189/jogh.16.04186

**Published:** 2026-07-24

**Authors:** Xiaokang Yao, Zishuo Wang, Tao Xiong, Mingrui Tao, Xiaotian Hu

**Affiliations:** 1 Division of Human Resources, The First People's Hospital of Yunnan Province, the Affiliated Hospital of Kunming University of Science and Technology, Kunming, Yunnan, China; 2 Business School, University of Jinan, Jinan, Shandong, China.; 3 Faculty of Basic Medical Science, Kunming Medical University, Kunming, Yunnan, China; 4 School of Public Health, Kunming Medical University, Kunming, Yunnan, China

**Keywords:** paediatric workforce, health workforce distribution, workforce sustainability, global health systems, medical subspecialties

## Abstract

**Background:**

The paediatric workforce plays a central role in health systems worldwide. National reports and studies have described its size and structure, as well as the retention of staff within the paediatric workforce. However, there have been few syntheses of empirical evidence examining workforce trends across career stages, and none have focused specifically on paediatric staff. We aimed to systematically synthesise evidence on the global trends of the availability, distribution, career progression, and differences across career stages among the paediatric workforce.

**Methods:**

We searched CENTRAL, Embase, MEDLINE, and Scopus between 1 and 20 November 2025 for primary studies reporting on the above-mentioned trends within the paediatric workforce. We extracted and narratively synthesised the related data, and assessed the risk of bias of included studies using Joanna Briggs Institute tools.

**Results:**

We included 35 studies from high-income and upper-middle-income countries, with no low- or lower-middle-income countries being eligible. Workforce supply increased steadily over time in high-income countries, driven largely by subspecialty growth. However, geographic imbalances persisted, with low representation of paediatric staff in rural areas. In terms of career stage, early-career paediatricians reported higher workload burden, dissatisfaction, and career uncertainty compared with their senior counterparts. Women progressively came to account for most of the workforce, although men continued to predominate in leadership positions. Evidence from upper-middle-income countries focused primarily on training preparedness and early-career experiences.

**Conclusions:**

Despite growth in the size of the paediatric workforce in high-income countries, challenges related to distribution, retention, and equity persist, particularly during early career stages. Importantly, the evidence base on this topic heavily underrepresents low- and lower-income countries. Future research should prioritise career-stage specific data to support equitable global workforce planning.

**Registration:**

PROSPERO: CRD420261283831.

The paediatric workforce is the backbone of health systems worldwide [1], with its training, equitable distribution, and retention being a recurring concern across both high- and middle-income countries [2,3]. Workforce shortages, uneven subspecialty growth, and imbalances in the distribution of providers among paediatric staff, however, raise questions about the sustainability of paediatric care and the ability of health systems to adapt to evolving child health needs [2,4–6].

Existing evidence on the paediatric workforce, however, has some limitations. Namely, most analyses have focused on the USA, resulting in poor representation of other geographical contexts. They have also been predominantly subspecialty-focused and reliant on certification records and survey data between the early 2000s and 2022 that capture only segments of paediatrics. Lastly, much of this research has been methodologically constrained, emphasising productivity measures such as hours worked or workforce equivalents, rather than broader workforce dynamics, and has been built largely on certification records and survey data [7,8]. Higher-level policy statements have outlined priorities for training, diversity, and equity, yet have been put forth as consensus documents, rather than systematic reviews and empirical syntheses [3].

Previous systematic reviews have primarily focused on the broader health workforce, including physician retention, health care worker job satisfaction, and workforce distribution [9–14]. For example, a systematic review of the maternal and child health workforce density in China [14] and which does not address paediatrics directly. This highlights the absence of a dedicated synthesis of paediatric workforce evidence across countries.

To address this gap, we sought to perform a systematic review of studies focusing on the training, career development, and retention trends within the paediatric workforce specifically, to identify what is known, which knowledge gaps remain, and how future research and policy can better support paediatric care in health systems worldwide.

## METHODS

We registered the protocol for this review in PROSPERO (CRD420261283831) and report it according to PRISMA guidelines [15].

### Eligibility criteria

We considered only observational studies (cross-sectional, longitudinal, repeated national surveys administrative data, and registry-based workforce analysis) conducted among licensed or trainee paediatricians at any career stages, including medical students who have selected paediatrics as their intended specialty, and early-career paediatricians (residents and registrars), irrespective of geographical contexts. Our primary outcomes were paediatric workforce availability/supply, distribution, and career dynamics; geographic workforce distribution; and career choice, retention, and transitions. The secondary outcomes were factors shaping paediatric career trajectories and sustainability, including workforce composition, equity, and diversity; work patterns, satisfaction, and well-being; and training preparedness and competence.

We excluded reviews, editorials, policy commentaries and statements, economic modelling, intervention studies, and documents without primary data (*e.g.* descriptive reports, expert opinion, *etc*.). We also excluded studies exclusively focusing on the non-physician health professional (*e.g.* pediatric nurses, physician assistants, and allied health staff). If any study reports multiple outcomes, we capture those based on our inclusion criteria and categorised as primary and secondary outcomes to avoid overlap.

### Search strategy

We searched MEDLINE (*via* PubMed), Embase, CENTRAL, and Scopus between 1 November and 20 November 2025, without temporal restrictions, using controlled vocabulary (MeSH/Emtree) and free text keywords related to paediatrics, workforce, career choice, training, competence, and satisfaction outcomes (Table S1 in the **Online Supplementary Document**). We also searched grey literature *via* Google Scholar using the same keywords and screened the reference lists of all included studies. We set no language restrictions. The search strategy was developed by the study team and reviewed for accuracy; however, it was not formally validated by an information specialist.

### Risk of bias assessment

Two reviewers (XY and ZW) independently assessed the methodological quality and risk of bias of the included studies using the Joanna Briggs Institute (JBI) critical appraisal tools [16]. Any conflict or disagreement was resolved through discussion, with a third reviewer (XH) adjudicating if consensus could not be reached.

### Data synthesis and analysis

Two researchers (XT and MT) independently extracted the following study data: author, year, country, and key findings, including workforce indicators, workforce growth, retention rates, workload measures, and training outcomes. Disagreements were resolved through discussion. Where possible, we standardised outcomes to improve comparability, expressing workforce density measures per 100,000 total population or per 100,000 children. We summarised categorical outcomes using odds ratios or risk ratios with 95% confidence intervals, and continuous outcomes using means or standardised mean differences. All eligible outcomes were extracted quantitatively wherever possible, with results reported using counts, proportions, rates, odds ratios, confidence intervals, or *P*-values, as appropriate.

Due to substantial heterogeneity in study populations, outcome measures, and designs, we did not perform a meta-analysis, but rather conducted a narrative synthesis following the Synthesis Without Meta-analysis framework [17]. Aside from performing a synthesis based on our pre-defined outcomes, we stratified our analysis by career stages, World Bank income groups, and geographic regions.

## RESULTS

**Figure 1:**
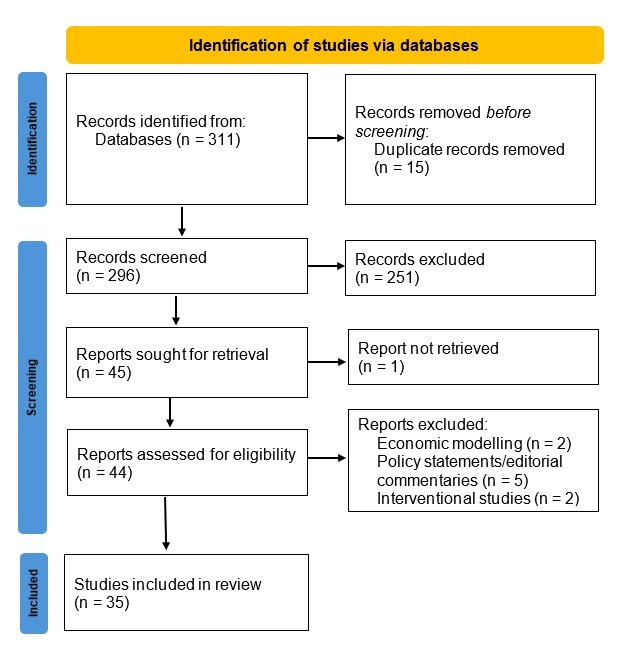
PRISMA flow diagram of study selection process.

We retrieved 311 records from the databases and, after removing 15 duplicates, screened 296 studies by title and abstract. We excluded 251 that did not address our outcomes of interest among the paediatric workforce and retrieved the full text of 45 articles [4–6,18–59]. One article [51] had no available full text; nine presented the results of economic modelling or intervention studies, or were policy statements/editorials [5,28,32,45,46,48,54,56,59], leaving 35 studies for synthesis [4,6,18–27,29–31,33–44,47,49,50,52,53,55,57,58] (**Figure 1**).

We included the 35 studies published between 1994 and 2025 representing eight countries from four continents (**Table 1**) [4,6,18–27,29–31,33–44,47,49,50,52,53,55,57,58]. Thirty studies came from North America (USA, Canada, and Mexico) [4,6,18–26,29,30,33–39,41–44,47,49,50,52,55,57] one from the South America (Brazil) [53], one from Asia (China) [40], and three from Europe (UK, France, and Italy) [27,31,58]. Furthermore, 17 studies were descriptive workforce reports (meaning they analysed existing secondary data sources, *e.g.* registries, administrative records, *etc*.) [4,6,18–26,47,50,52,53,55,57], 15 were cross-sectional [27,29–31,33,34,36–38,40–44,49], two adopted a retrospective cohort design [39,58], and one had a repeated cross-sectional design [35]. Lastly, 23 studies reported on multiple outcomes [4,6,18–26,30,33–36,44,47,52,53,55,57,58] and 14 on a single outcome [27,29,31,37–43,49,50].

**Table 1:** Characteristics of the included studies

Author (year)	Country	Continent	Setting	Study design	Data source	Findings	Career stage	Overall sample size
Althouse and Stockman (2006) [19]	USA, Canada	North America	Paediatric residency and certification programmes	Descriptive observational workforce study	ABP tracking system, questionnaires, certification database	Increase in paediatric residents over time; women comprise 68.9% of entering residents; >60% select general paediatrics; subspecialty interest increased from 20% (1998) to 25% (2004); national paediatrician-to-child ratio ≈ 1:1700; geographic maldistribution across states	Residents, fellows, certified paediatricians	82,270 certified historically
Althouse and Stockman (2006) [20]	USA, Canada, Puerto Rico	North America	Neonatal-perinatal medicine training programmes and certified workforce	Descriptive observational workforce study	American Board of Pediatrics tracking system, examination application surveys, certification database	Number of neonatal-perinatal fellows increased by 47.7% since 1997; women comprise 57.8% of fellows; AMG proportion increased to 56.3%; subspecialty interest reported by 18% of subspecialty-interested residents; ≈ 4500 certified neonatologists; national ratio 5.1 per 100,000 children with large state variation	Fellows; certified subspecialists	≈4,500 certified neonatologists
Althouse and Stockman (2006) [21]	USA, Canada	North America	Paediatric cardiology training programmes and certified workforce	Descriptive observational workforce study	American Board of Pediatrics tracking system, examination application surveys, certification database	Number of paediatric cardiology fellows increased by 40.6% since 1997; average training drop rate 16.7%; cardiology selected by 10% of subspecialty-interested residents; ≈ 1850 certified historically; national ratio ≈ 1.8 cardiologists per 100,000 children with marked state variation	Fellows; certified subspecialists	>1850 certified historically
Althouse and Stockman (2006) [22]	USA, Canada, Puerto Rico	North America	Paediatric critical care medicine training programmes and certified workforce	Descriptive observational workforce study	American Board of Pediatrics tracking system, examination application surveys, certification database	Number of fellows increased by 40.8% since 1997; women fellows increased to 44.6%; average drop rate 20%; critical care selected by 8.4% of subspecialty-interested residents; ≈ 1300 certified physicians; national ratio ≈ 1.6 per 100,000 children with marked state variation	Fellows; certified subspecialists	≈ 1300 certified historically
Althouse and Stockman (2006) [23]	USA, Canada	North America	Paediatric gastroenterology training programmes and certified workforce	Descriptive observational workforce study	American Board of Pediatrics tracking system, examination application surveys, certification database	Number of fellows increased by 80% since 1997; women fellows currently 42.5%; average dropout rate 16%; gastroenterology selected by 6.2% of subspecialty-interested residents; ≈ 900 certified gastroenterologists; national ratio ≈ 1.1 per 100,000 children with marked state variation	Fellows; certified subspecialists	≈900 certified historically
Althouse and Stockman (2006) [24]	USA, Canada	North America	Paediatric rheumatology training programmes and certified workforce	Descriptive observational workforce study	American Board of Pediatrics tracking system, examination application surveys, certification database	Number of fellows more than doubled since 1997; women fellows currently 64.6%; average drop rate 21%; rheumatology selected by 2.7% of subspecialty-interested residents; ≈215 certified rheumatologists; national ratio ≈0.3 per 100,000 children with severe geographic maldistribution	Fellows; certified subspecialists	≈215 certified historically
Althouse and Stockman (2007) [25]	USA, Canada	North America	Paediatric infectious diseases training programmes and certified workforce	Descriptive observational workforce study	American Board of Pediatrics tracking system, examination application surveys, certification database	Number of fellows more than doubled since 1997; women comprise 58.1% of fellows; average drop rate 13%; infectious diseases selected by 6% of subspecialty-interested residents; ≈1000 certified physicians; national ratio ≈1.2 per 100,000 children with substantial geographic variation	Fellows; certified subspecialists	≈1000 certified historically
Althouse and Stockman (2011) [26]	USA, Canada	North America	Paediatric residency and subspecialty training programmes and certified workforce	Descriptive observational workforce study	American Board of Pediatrics tracking system, ITE/SITE, examination application surveys, certification database	Growth in general paediatrics and subspecialty workforce; 7.5% increase in categorical paediatrics since 2004–2005; women now 73.2% of entering residents; subspecialty interest increased to 33%; subspecialty trainees increased 32.3% since 2004–2005; national paediatrician-to-child ratio improved to ~1:1,400	Residents; fellows; certified paediatricians	99,258 certified paediatricians (historical)
Anne *et al*. (2007) [27]	France	Europe	Six university hospitals (Western Interregion)	Cross-sectional survey study	Telephone questionnaire of former paediatric residents	77% felt training was adapted to current practice; satisfaction higher in hospital paediatricians (82%) than private (50%); 86% satisfied with professional life; 73% satisfied with personal life; 75% completed or were completing post-residency training	Former paediatric residents (DES)	187 eligible; 165 respondents
Cull *et al*. (2002) [29]	USA	North America	National paediatric practice and residency workforce	Cross-sectional survey study (repeated cross-section + resident survey)	AAP Periodic Surveys (1993, 2000); national survey of graduating paediatric residents (2000)	Number of part-time paediatricians increased from 11% (1993) to 15% (2000); women far more likely to work part-time than men (26% vs 4%); part-time paediatricians provide ~36% fewer direct patient-care hours; female residents show markedly higher interest in future part-time work	Practicing paediatricians; third-year paediatric residents	Practicing paediatricians: 1,536 (1,993 + 2,000); residents: 354
Cull *et al*. (2003) [30]	USA	North America	Paediatric residency programmes nationwide	Repeated cross-sectional national survey (trend analysis)	AAP Third-Year Resident Survey (1997–2002)	Female and URM residents increased; IMG proportion decreased; educational debt rose sharply; interest in general paediatrics declined, while subspecialty interest increased; starting salaries in general paediatrics declined; job market tightened for general paediatrics and improved for subspecialty training	Third-year paediatric residents	~3,000 surveyed across 6 years
Del Bene *et al*. (2025) [31]	Italy	Europe	Primary care paediatrics, emergency departments, hospital wards	Cross-sectional national survey	Google Forms survey of paediatric residents and tutors	Only 3% of first-year residents felt fully autonomous in ED/HW; autonomy increased progressively with year, reaching 72% (ED) and 83% (HW) by year 5; PCP autonomy highest across all years (15% by year 1 to 96% by year 5); no significant differences between resident self-evaluation and tutor evaluation	Residents; tutors	391 total respondents
Duvivier *et al*. (2020) [4]	USA	North America	National paediatric workforce	Cross-sectional population study	AMA Physician Masterfile (2019); ECFMG database	IMGs constitute 23.2% of the paediatric workforce; 22.1% of IMGs are US citizens; IMGs are older, more likely to work in solo practice, and less likely to work in group or hospital-based practice; substantial state-level variation with IMGs comprising up to ~48% of paediatricians in some states	Residents; fellows; practicing paediatricians	92,806 paediatric physicians
Freed (2006) [33]	USA	North America	Paediatric and internal medicine residency graduates	Cross-sectional mail survey	ABP and American Board of Internal Medicine graduate lists	Categorically trained paediatricians felt better prepared for infant care but less prepared for adolescent care than medicine-paediatrics physicians; categorically trained internists felt less prepared for adult and elder care than med-peds; differences consistent across graduation cohorts	Practicing paediatric physicians	596 practicing physicians
Freed *et al*. (2009) [34]	USA, Canada	North America	Paediatric residency programmes	Cross-sectional national survey	ABP General Paediatrics In-Training Examination add-on survey (2007)	95% response rate; ~47–49% of residents planned fellowship training across all years; lifestyle most important factor for general paediatrics (63% *vs*. 21% in subspecialties); location most important residency selection factor (42%); women and AMGs more likely to pursue general paediatrics	Residents (PGY-1 to PGY-3)	7,882 respondents
Freed *et al*. (2009) [35]	USA	North America	General paediatrics practice (post-residency)	Cross-sectional national mail survey	ABP certification database	62% reported need for more mental health training; >50% desired more training in sports medicine and oral health; 83% had local access to subspecialists; generalists without subspecialist access reported greater comfort managing subspecialty conditions independently	Recently trained general paediatricians (1–2 years *vs*. 4–5 years post-training)	685 general paediatricians
Freed *et al*. (2015) [36]	USA	North America	Early general paediatrics workforce	Cross-sectional national survey	ABP General Paediatrics Certifying Examination add-on survey (2012)	99% response rate; lifestyle and family considerations most important factor for first job; 83% reported current job matched desired allocation of professional time; majority desired outpatient-focused practice with minimal inpatient responsibilities	Newly trained general paediatricians	5163 respondents
Green *et al*. (2020) [19]	USA	North America	Paediatric residency programmes	Cross-sectional national survey with multivariable analysis	ABP Initial Certifying Examination applicant survey (2018)	Only 32.8% of trainees reported high assessment competence and 18.9% high treatment competence in B/MH; substantial programme-level variation exists; trainees from smaller programmes report significantly higher competence even after multivariable adjustment	Residents; recent graduates	2,086 respondents
Gustafson *et al*. (2024) [38]	USA	North America	Academic paediatrics	Narrative review	Published literature; AAMC; ACGME; ABP; census and survey data	Structural racism contributes to persistent underrepresentation of URiM trainees and faculty in paediatrics; URiM representation has stagnated among residents and declined among fellows; disparities exist in dismissal rates, mentorship, and faculty retention	Residents; fellows; faculty	NA
Kurahara *et al*. (2020) [39]	USA (Hawai‘i)	North America	Single paediatric residency programme	Retrospective cohort study with multivariable logistic regression	University of Hawai‘i Paediatric Residency Programme alumni database (1968–2015)	56.7% of graduates remained in Hawai‘i; graduating from the local medical school strongly predicted in-state retention (aOR = 7.46); pursuing specialty training reduced odds of in-state practice (aOR = 0.38); female residents were significantly more likely to choose general paediatrics (aOR = 3.05)	Residency graduates	319 graduates
Li *et al*. (2024) [40]	China	Asia	Two tertiary children’s hospitals (Shanghai)	Mixed-methods (cross-sectional survey + qualitative interviews)	Jefferson Scale of Empathy–Student Version (JSE-S); semi-structured interviews	Mean empathy score was low at 81.41 (SD = 5.43); no significant differences by gender, specialty, education level, or residency year; work environment and workload strongly influenced empathy; residents valued empathy but reported minimal formal training	Residents (PGY1–PGY3)	154 survey respondents
Li *et al*. (2023) [41]	USA	North America	Paediatric residency graduates entering workforce	National cross-sectional survey with multivariable logistic regression	ABP Initial General Paediatrics Certifying Examination survey (2021)	29.1% reported COVID-19 impacted their career; impact highest among those seeking GP or paediatric hospitalist jobs; IMGs significantly more affected; COVID-19 influenced career choice, job search, and employment offers	Residents, chief residents, recent graduates	1,767 respondents
Lieberman and Hilliard (2006) [42]	Canada	North America	Canadian paediatric residency programmes	Cross-sectional national survey	RCPSC certification list (1999–2003 graduates)	96% felt adequately or very well trained; strongest preparation in acute care and subspecialties; weakest preparation in gynecology, child psychiatry, behavioral paediatrics, adolescent medicine, and office practice management; 80% felt four years of training was sufficient	Practicing paediatricians (recent graduates)	239 respondents
Althouse and Stockman (2006) [22]	USA, Canada	North America	Paediatric endocrinology workforce	Descriptive workforce report	ABP master database; fellow tracking; certification application surveys	Paediatric endocrinology workforce expanded substantially; 122% increase in fellows since 1997; women comprise ~73% of trainees; workforce maldistribution persists with many states below 1 endocrinologist per 100,000 children	Fellows; certified paediatric endocrinologists	Not a single cohort
Freed *et al*. (2016) [6]	USA	North America	Practicing general paediatricians	National cross-sectional survey with multivariable logistic regression	ABP Maintenance of Certification (MOC) enrollment survey (2013–2014)	25% of general paediatricians work part-time; women had dramatically higher odds of part-time work (OR = 12.21); most paediatricians reported their job matched desired duties; QI participation associated with full-time work and non-private practice; retirement intentions varied by sex, IMG status, and years since training	Practicing paediatricians	9,253 general paediatricians
Minkovitz *et al*. (2006) [43]	USA	North America	Paediatric residency programmes	Cross-sectional national survey with multivariable linear regression	AMA Physician Masterfile national resident sample (2001–2002)	Women reported greater prior exposure to community activities, rated community training as more important, and anticipated greater future community involvement; gender remained independently associated with anticipated future involvement in five community settings after adjustment	Residents (PL1–PL3)	700 residents
Minkovitz *et al*. (2007) [44]	USA	North America	Practicing paediatricians	National cross-sectional survey	American Academy of Paediatrics Periodic Survey 60 (2004)	Younger paediatricians reported more community health training but less current involvement; they perceived their involvement as insufficient and expected future increases; age differences persisted after adjustment for gender	Practicing paediatricians	876
Pearson (1994) [47]	USA	North America	National paediatric workforce policy	Consensus policy statement	Expert consensus (Federation of Paediatric Organizations)	Identified national shortage and maldistribution of paediatricians; emphasized need to expand primary care paediatrics; recommended creation of a National Health Care Workforce Commission; advocated protection of IMG contributions, incentives for primary care training, and restructuring of GME funding	NA	NA
Sepúlveda-Vildósola *et al*. (2006) [49]	Mexico	North America	Hospital de Pediatría, Centro Médico Nacional Siglo XXI (IMSS)	Cross-sectional single-center survey with mixed quantitative–qualitative analysis	Original resident survey (Likert scales + semantic networks)	Prevalence of dissatisfaction with paediatrics training was 3.1%; dissatisfaction concentrated in first two years; no sociodemographic variables were statistically associated; main drivers were educational strategies, workload, hierarchy, abuse, and long working hours	Residents (PGY1–PGY4)	64 residents
Shah and Cheng (2022) [50]	USA	North America	Paediatric workforce and GME financing	Policy analysis / narrative review	Secondary sources (ABP, AAP, CHA, CRS, BLS, ChildStats)	Describes persistent shortages and maldistribution of paediatric subspecialists; outlines declining real-term CHGME funding per trainee; argues that inadequate and unstable GME funding contributes to subspecialty access problems and recommends multiyear, indexed CHGME funding increases	NA	NA
Shipman *et al*. (2004) [52]	USA	North America	National general paediatric workforce	Quantitative workforce projection model with sensitivity analyses	AMA and AOA Masterfiles; AMA GME Survey; US Census; NAMCS; AAP surveys	General paediatrician supply projected to grow 58–64% by 2020 while child population grows ~9%; paediatricians per child increase substantially under all plausible scenarios; even major reductions in GME or productivity do not produce shortages	Practicing paediatricians (modeled)	NA
Silva *et al*. (2021) [53]	Brazil	South America	University paediatric department graduates	Cross-sectional online survey	REDCap survey of paediatricians graduating 2006–2018	Early-career paediatricians (≤5 years) reported higher workload, lower income, more work-related issues (long hours, poor social life, harassment), and lower satisfaction with residency compared with those >5 years; overall satisfaction with clinical practice remained high	Practicing paediatricians (post-residency)	331 respondents (54% response rate)
Spector *et al*. (2014) [55]	USA	North America	Paediatric workforce (national)	Special article: narrative review with limited original survey analyses	FOPO working group synthesis; AAP Periodic Survey; original survey of paediatric department chairs	The increasing proportion of women entering paediatrics is not associated with reduced workforce capacity; men and women practice paediatrics similarly; women are less likely to enter research careers; generational shifts toward flexibility, part-time work, and technology adoption will significantly reshape paediatric practice	Residents; practicing paediatricians; department chairs	Multiple secondary data sets
Tunnessen *et al*. (2001) [57]	USA and Canada	North America	National paediatric workforce	Descriptive longitudinal workforce analysis (administrative data)	American Board of Paediatrics resident and fellow tracking; certifying exam questionnaires	Paediatric residents increased 30.7% from 1991–2000; proportion of women increased markedly; interest in subspecialties declined during the 1990s then rebounded slightly by 2000; IMG participation decreased; fellowship attrition remained low (~5.5%)	Residents; fellows; exam candidates	National census
Turner *et al*. (2007) [58]	United Kingdom	Europe	National medical graduate workforce	Repeated national postal surveys with longitudinal follow-up to 10 y	UK Medical Careers Research Group cohort surveys (1974–2002 graduates)	~7% of UK graduates chose paediatrics 1 year after graduation; women were twice as likely as men to choose paediatrics; only 44% (year 1 choosers) and 62% (year 3 choosers) were working in paediatrics 10 years later; enthusiasm and undergraduate experience were key drivers	Medical graduates (1 year, 3 years, and 10 years post-graduation)	Year 1: 24,621; Year 3: 20,720

AAMC – Association of American Medical Colleges, AAP – American Academy of Pediatrics, ABP – American Board of Pediatrics, ACGME – Accreditation Council for Graduate Medical Education, AMG – American medical graduate, AOA – American Osteopathic Association, aOR – adjusted odds ratio, B/MH – behavioral/mental health, BLS – Bureau of Labor Statistics, CHA – Children's Hospital Association, CHGME – Children's Hospitals Graduate Medical Education, DES – Diplôme d'Études Spécialisées (French specialist diploma), ECFMG – Educational Commission for Foreign Medical Graduates, ED – emergency department, FOPO – Federation of Pediatric Organizations, GME – graduate medical education, IMG – international medical graduate, ITE – in-training examination, MOC – maintenance of certification, NAMCS – National Ambulatory Medical Care Survey, OR – odds ratio, PCP – primary care pediatrics, PGY – postgraduate year, RCPSC – Royal College of Physicians and Surgeons of Canada, SD – standard deviation, SITE – subspecialty in-training examination, URM – underrepresented minority, URiM – underrepresented in medicine

### Risk of bias assessment

The included studies had low and moderate risk of bias, with variations in sampling strategies, response rates, and analytical depth (**Table 2**). Most studies employed descriptive analytical approaches and only a few used inferential statistics and adjusted for confounders. The certainty of the evidence was highest for the descriptive outcomes related to workforce size and distribution, and was low for outcomes based on self-reported perceptions, including preparedness, satisfaction, and well-being. We could not apply the JBI tools to two descriptive workforce reports [47,50] because they were based on secondary data.

**Table 2:** Risk of bias assessment using the Joanna Briggs Institute (JBI) critical appraisal tools

Author (year)	Sampling frame	Sampling method	Response rate	Missing data	Confounding identified	Confounding addressed	Statistical analysis	Overall
Althouse and Stockman (2006) [18]	ABP residents, fellows, certified paediatricians	Census-style administrative tracking	~94% residents; ~64% fellows; ~100% programme participation	Not specified	Yes (gender, geography, work hours)	No	Descriptive statistics only	Moderate
Althouse and Stockman (2006) [19]	ABP neonatal-perinatal fellows and certified diplomates	Census-style administrative tracking	100% programme participation (108/108 programmes)	Minimal (1 fellow missing AMG/IMG data)	Yes (geography, part-time work, gender)	No	Descriptive statistics only	Moderate
Althouse and Stockman (2006) [20]	ABP paediatric cardiology fellows and certified diplomates	Census-style administrative tracking	100% programme participation (55/55 programmes)	Minimal	Yes (geography, workload, part-time work)	No	Descriptive statistics only	Moderate
Althouse and Stockman (2006) [21]	ABP paediatric critical care fellows and certified diplomates	Census-style administrative tracking	100% programme participation (70/70 programmes)	Minimal	Yes (gender, geography, workload, part-time work)	No	Descriptive statistics only	Moderate
Althouse and Stockman (2006) [23]	ABP paediatric gastroenterology fellows and certified diplomates	Census-style administrative tracking	~98% programme participation (52/53 programmes)	Minimal	Yes (gender, geography, work patterns)	No	Descriptive statistics only	Moderate
Althouse and Stockman (2006) [24]	ABP paediatric rheumatology fellows and certified diplomates	Census-style administrative tracking	~93% programme participation (26/28 programmes)	Minimal	Yes (gender, geography, workforce size)	No	Descriptive statistics only	Moderate
Althouse and Stockman (2007) [25]	ABP paediatric infectious diseases fellows and certified diplomates	Census-style administrative tracking	100% programme participation (69/69 programmes)	Minimal	Yes (gender, geography, workload)	No	Descriptive statistics only	Moderate
Althouse and Stockman (2011) [26]	ABP residents, fellows, and certified paediatricians	Census-style administrative tracking	~100% programme participation; ~94% residents (ITE); 64–83% fellows (SITE)	Minimal	Yes (sex, geography, part-time work, specialty choice)	No	Descriptive statistics only	Moderate
Anne *et al*. (2007) [27]	Former paediatric residents (1990–2000 cohorts)	Census of eligible graduates	88%	Minimal	Yes (practice setting, gender, workload)	No	Descriptive statistics only	Moderate
Cull *et al*. (2002) [29]	AAP paediatricians and graduating paediatric residents	National random samples	Practicing paediatricians: 62% overall; Residents: 71%	Some item-level missingness	Yes (gender, age, family factors)	Partially (multivariable models)	χ^2^ tests, *t-*tests, ANOVA, logistic regression	Moderate
Cull *et al*. (2003) [30]	National third-year paediatric residents	Annual national random samples	71% overall (64%–78% by year)	Some item-level missingness	Yes (gender, IMG status, career goal, year)	Partially (trend regression)	Linear regression; χ^2^ trend tests	Moderate
Del Bene *et al*. (2025) [31]	Italian paediatric residents and tutors nationwide	Voluntary web-based survey	Residents ≈9%; tutors unknown	Minimal item-level missingness	Yes (year, setting, region)	No	χ^2^ tests, Fisher exact tests	Moderate to high
Duvivier *et al*. (2020) [4]	Entire US paediatric workforce (AMA + ECFMG)	Census of active physicians	Not applicable	Practice type missing for ~19% IMGs	Yes (age, sex, practice type)	Not applicable (descriptive census)	Descriptive statistics only	Low to moderate
Flores *et al*. (2021) [32]	RAPID applicants nationwide	Census of applicants with longitudinal follow-up	84% baseline; 71–83% follow-up	Some attrition over time	Yes (baseline productivity, mentorship, motivation)	Partially (comparison groups)	Descriptive statistics only	Moderate
Freed (2006) [33]	ABP & ABIM certified physicians	National random samples	Paediatricians 78%; Internists 64%	Minimal	Yes (graduation cohort, specialty)	No	Descriptive comparisons; χ^2^ tests	Moderate
Freed *et al*. (2009) [34]	All US and Canadian paediatric residents	Census via in-training exam	95%	Minimal	Yes (gender, IMG, programme size, PGY)	No	Descriptive statistics; χ^2^ tests	Moderate
Freed *et al*. (2009) [35]	ABP-certified general paediatricians	National random sample	76%	Minimal	Yes (gender, access to subspecialists)	No	Descriptive statistics; χ^2^ tests	Moderate
Freed *et al*. (2015) [36]	ABP certifying-exam candidates	Census via exam add-on survey	99%	Minimal	Yes (gender, time since training)	No	Descriptive statistics; χ^2^ tests	Moderate
Green *et al*. (2020) [37]	US paediatric residency trainees	Census of exam applicants	62.30%	Minimal	Yes (programme size, demographics)	Yes (multivariable regression)	Linear regression with CIs	Moderate
Gustafson *et al*. (2024) [38]	Not applicable	Narrative synthesis	Not applicable	Not applicable	Yes (structural racism, gender, rank)	No	Descriptive synthesis only	Moderate
Kurahara *et al*. (2020) [39]	All graduates of one residency programme	Complete cohort	Not applicable	Minimal (9 unknown locations)	Yes (sex, school, specialty, year)	Yes (multivariable logistic regression)	Logistic regression with ORs and CIs	Moderate
Li *et al*. (2024) [40]	Paediatric residents at two hospitals	Voluntary online survey	85.10%	Minimal	Yes (sex, PGY, specialty, education)	No	*t*-tests; Mann-Whitney; ANOVA	Moderate
Li *et al*. (2023) [41]	ABP exam registrants	National census survey	52.30%	Minimal	Yes (career plan, IMG status, debt, region)	Yes	Multivariable logistic regression with ORs and 95% CIs	Moderate
Lieberman and Hilliard (2006) [42]	RCPSC-certified paediatricians (1999–2003)	National mail survey	55%	Minimal	Yes (practice type, setting)	No	Descriptive statistics; χ^2^ tests	Moderate
Althouse and Stockman (2006) [22]	ABP-tracked fellows and certified endocrinologists	Census of administrative data	97% programme response	Minimal	Yes (sex, IMG status, geography)	No	Descriptive statistics only	Moderate
Freed *et al*. (2016) [6]	ABP-certified paediatricians in MOC	National census survey	87.20%	Minimal	Yes (sex, IMG, academic role, practice type)	Yes	Multivariable logistic regression with ORs and 95% CIs	Low–Moderate
Minkovitz *et al*. (2006) [43]	US paediatric residents	National random sample	43%	Low	Yes (prior exposure, programme size, URM status)	Yes	χ^2^ tests, ANOVA, multivariable linear regression	Moderate
Minkovitz *et al*. (2007) [44]	US AAP member paediatricians	National random mailed survey	57.60%	Low	Yes (age, gender)	Partial (gender-adjusted age analyses)	χ^2^ tests; Kruskal-Wallis median tests	Moderate
Pearson (1994) [47]	Not applicable	Not applicable	Not applicable	Not applicable	Not applicable	Not applicable	None	Not applicable
Sepúlveda-Vildósola *et al*. (2006) [49]	Paediatric residents at a tertiary hospital	Census of eligible residents	74.40%	Low	Yes (age, sex, PGY)	No	Descriptive statistics; χ^2^ tests; Spearman correlation	Moderate
Shah and Cheng (2022) [50]	Not applicable	Not applicable	Not applicable	Not applicable	Not applicable	Not applicable	None	Not applicable
Shipman *et al*. (2004) [52]	National paediatric workforce	Modeled using secondary data sets	Not applicable	Not applicable	Yes (demographics, GME, productivity)	Sensitivity analyses	Deterministic projection modeling	Moderate
Silva *et al*. (2021) [53]	Paediatric residency graduates (single institution)	Census with email invitations	54%	Moderate	Yes (career stage, age)	Partial (stratification only)	Descriptive statistics; χ^2^ tests; Mann-Whitney tests	Moderate
Spector *et al*. (2014) [55]	Paediatric leaders & secondary data sets	Mixed (survey + literature synthesis)	Chairs survey: 70%	Not specified	Yes (gender, age)	Limited (stratification only)	Descriptive and Fisher exact tests	Moderate
Stefanski *et al*. (2025) [56]	PGY-1 paediatric residents at one institution	Census of eligible residents	97%	Minimal	Yes (prior exposure, career intent)	No	Bhapkar’s test; descriptive statistics	Moderate
Tunnessen *et al*. (2001) [57]	National paediatric trainees & examinees	Near-complete census	~100%	Minimal	Not applicable	Not applicable	Descriptive statistics only	Low
Turner *et al*. (2007) [58]	All UK medical graduates (selected cohorts)	National census-based surveys	~73–74%	Low	Yes (sex, cohort, school)	Partial (stratification, regression)	χ^2^ tests; Wald statistics	Low to moderate

AAP – American Academy of Pediatrics, ABIM – American Board of Internal Medicine, ABP – American Board of Pediatrics, AMA – American Medical Association, ANOVA – analysis of variance, χ^2^ – chi-square test, CI – confidence interval, ECFMG – Educational Commission for Foreign Medical Graduates, IMG – international medical graduate, ITE – in-training examination, MOC – maintenance of certification, OR – odds ratio, PGY – postgraduate year, RAPID – Research in Academic Pediatrics Initiative on Diversity, RCPSC – Royal College of Physicians and Surgeons of Canada, SITE – subspecialty in-training examination, URM – underrepresented minority

### Primary outcomes

#### Workforce availability and supply

Evidence on workforce availability and supply was dominated by large administrative and registry-based studies, primarily from high-income countries. The paediatric workforce expanded steadily from the 1990s through the early 2010s, driven by residency training and subspecialty certification [18–26,47,57]. Subspecialty growth was particularly pronounced in neonatal–perinatal medicine, cardiology, critical care, and infectious diseases, while growth in general paediatrics appeared to slow in later years [18,26]. Although workforce numbers increased overall, several studies noted that growth did not occur uniformly across subspecialties or geographic regions, raising concerns about potential imbalances between general paediatric practice and subspecialty care. Recent data suggest the overall supply remains robust, but increasingly reliant on subspecialists, rather than general paediatricians, raising concerns about generalisability beyond the USA, as our analysis relies primarily on American Board of Pediatrics datasets [6,50,55].

#### Geographic and workforce distribution

Despite overall workforce growth, imbalances in its geographic distribution persisted over decades. Studies consistently reported higher paediatrician density in urban and academic centers, while rural and peripheral regions remained undersupplied [6,26,52]. Career stage and background played an important role in shaping practice location. Early-career paediatricians were less likely to practice in rural settings, while graduates with local or regional training ties showed higher retention within the same state or region [39,58]. This suggests that supply increases alone do not resolve geographic inequities without targeted policy interventions.

#### Career choice, retention, and transitions

Fifteen studies examined career choice, retention, or transitions across the paediatric career pipeline. Women more commonly entered into paediatrics than men, though interest varied by country and cohort [27,33–35,58]. Longitudinal and repeated cross-sectional studies highlighted substantial attrition over time. In the UK, fewer than half of graduates who initially selected paediatrics were practicing in the specialty ten years later, with retention improving among those who reaffirmed their choice several years after graduation [58]. In the USA, early-career paediatricians reported actively reconsidering their long-term career trajectories, particularly during their transition from residency to independent practice [34–36,53]. Most recent cohorts entering the workforce during the COVID-19 pandemic reported altered career expectations, with evidence that 42% of residents graduating in 2021 were reconsidering their long-term career plans compared with 28% in pre-pandemic cohorts [41].

### Secondary outcomes

#### Workforce composition, equity, and diversity

Fourteen studies addressed workforce composition, with most focusing on gender differences. Across multiple data sets, the workforce became progressively feminised, with women forming the majority of trainees and practitioners, rising from ~45% in the 1990s to >65% in recent cohorts [6,55,57]. However, representation was uneven across career stages and leadership roles. Senior academic and leadership positions remained disproportionately occupied by men, despite the feminisation of the trainee pipeline [55]. Fewer studies focused on racial and ethnic diversity; those that did highlighted persistent structural barriers and slower advancement for underrepresented groups within paediatrics [4,38].

#### Work patterns, satisfaction, and well-being

Thirteen studies examined work patterns, job satisfaction, or well-being. Early-career paediatricians and residents consistently reported heavier workloads, greater dissatisfaction, and higher levels of stress compared with more senior colleagues [29,49,53]. Part-time work increased over time and was more common among women, reflecting both changing workforce preferences and broader shifts in work–life balance expectations. For instance, 32% of female paediatricians worked part-time compared with 12% of male colleagues [29,43,44]. While many paediatricians reported overall job satisfaction, concerns related to workload intensity, administrative burden, and scope of practice were common, particularly among those in their first years of independent practice [36,53].

#### Training preparedness and competence

Nine studies focused on training preparedness and perceived competence. Across settings, most residents felt well prepared for core inpatient paediatrics, but confidence varied by clinical domain [27,42]. Simultaneously, workforce data showed that the number of fellows entering subspecialty training programmes increased substantially between the late 1990s and mid-2000s, while the number of general paediatricians grew at a slower pace. Later workforce analyses suggested that overall paediatric workforce supply remained stable, but increasingly relied on subspecialty-trained physicians [6,55].

There were consistent gaps in behavioral and mental health care, community paediatrics, and the management of complex psychosocial problems [31,37]. Preparedness and autonomy increased with training seniority, yet even senior trainees reported uncertainty in managing patients independently in certain settings [31,40,42]. Studies examining training experiences reported that perceived competence increased with training seniority, although gaps remained in areas such as behavioral health and community paediatrics.

### Cross-cutting patterns across outcomes

Three consistent themes emerged across domains: workforce growth did not resolve the challenges of imbalanced distribution or retention; career stage strongly shaped experiences, with early-career paediatricians facing the greatest pressures; and evidence was strongest for workforce size and composition, but weaker for long-term outcomes such as retention, well-being, and career progression.

## DISCUSSION

In this systematic review, we synthesised evidence from 35 empirical studies examining global trends in the paediatric workforce, focusing on career stage, workforce distribution, and training outcomes. Paediatricians were concentrated in urban centers, while rural regions remained undersupplied. Local training ties improved retention somewhat, but inequities persisted. Paediatrics was a popular specialty choice among women, yet leadership positions remained disproportionately male. Early-career paediatricians consistently reported heavier workloads, greater stress, and lower satisfaction compared with senior colleagues. Part-time work, more common among women, increased over time, reflecting evolving workforce preferences. Training was generally adequate for inpatient paediatrics, but showed persistent gaps in behavioral health, community paediatrics, and complex psychosocial care. Targeted interventions improved short-term confidence, though long-term effects were uncertain. Overall, workforce expansion did not resolve challenges of maldistribution, retention, or equity, and evidence on long-term outcomes such as well-being and career progression remains limited.

Although interventions such as structured training exposures, mentorship programmes, and retention incentives have been reported in the broader workforce literature, they were not relevant for our review, which focused on observational studies. As a result, our synthesis highlights the absence of robust intervention evidence specific to paediatrics, underscoring the need for future studies that evaluate the effectiveness of strategies in addressing the imbalanced distribution of the paediatric workforce, early-career burnout, and training deficiencies.

### Agreements and disagreements with systematic reviews

To our knowledge, no systematic review has previously focused specifically on the paediatric specialty, so our findings can only be related to analogous reviews in the broader health workforce. Reviews of nurse and physician retention emphasise social support and work–life balance as critical to sustainability [60,61], which aligns with our findings on early-career paediatricians facing heavy workloads and uncertainty. Reviews of integrated care highlight working conditions and interpersonal relationships as determinants of job satisfaction [62], yet they did not address paediatric-specific challenges as we did in our review. Systematic reviews of career intentions among health science students report persistent barriers to paediatrics, including perceptions of low prestige and demanding hours [63], which our findings confirm and extend by linking them to long-term workforce imbalances. Reviews of maternal health workforce interventions in low- and middle-income countries underscore the importance of supportive environments and career development [12], while our synthesis reveals a striking absence of empirical paediatric workforce studies from such contexts. In sum, our findings are broadly consistent with previous health workforce reviews that highlight workload, work–life balance, and professional support as key determinants of physician retention.

### Quality of evidence assessment

The studies included in this review were mostly cross-sectional in design, with very few longitudinal investigations tracking career trajectories or retention over time. They predominantly came from high-income and, to a lesser extent, upper-middle income settings, with no empirical evidence from the low- or lower-middle-income countries. This represents a blind spot in the global literature, particularly as paediatric workforce shortages are most severe in these settings [64,65], which presents a priority for future research and policy development. The overall bias for the included studies ranged from low to moderate, with higher certainty for descriptive outcomes (*e.g.* workforce size and distribution) and lower certainty for self-reported ones (*e.g.* preparedness, satisfaction, and well-being).

### Study limitations

Several limitations of this review should be noted. First, most of the studies came from high-income countries, which limits the generalisability of our findings to other contexts. Second, while we designed an extensive search strategy for this review and performed dual screening, it remains possible that we overlooked some articles and unpublished data, as our grey literature search was limited to Google Scholar only. To mitigate this, we also ran searches in Google Scholar using the same keywords we used in the primary database search. Third, the predominance of cross-sectional designs restricts insight into long-term workforce trends. Fourth, the focus on subspecialty paediatrics in many studies may have led us to overlook evidence on general paediatrics and community-based care. Lastly, the studies in our sample were deemed to be at low or moderate risk of bias, which constrained the certainty of the evidence in our analysis.

While the evidence base remains geographically concentrated, uneven in design, and disproportionately focused on subspecialty paediatrics, our review contributes by providing the first global synthesis of paediatric workforce research across career stages. By systematically mapping gaps in general paediatrics, community practice, and leadership pathways, we highlight priority areas for future investigation. For policymakers, the findings underscore the need to address inequitable distribution of paediatricians, strengthen retention strategies for early‑career practitioners, and ensure sustainable career pathways that balance subspecialty growth with community‑based care. In doing so, this review offers an evidence‑informed foundation for workforce planning and policy development in child health systems.

## CONCLUSIONS

The global paediatric workforce has expanded over recent decades, yet important challenges remain related to workforce distribution, career retention, and evolving work patterns. Future workforce planning efforts should prioritise strategies that address geographic disparities, support early-career physicians, and strengthen training programmes to meet emerging health care needs. Further research, however, is needed to better understand paediatric workforce dynamics in low- and middle-income countries and to identify effective policy interventions that promote sustainable workforce development.

## Additional material


Online Supplementary Document
