# Supplementary material for: Global trends and career development of human resources for paediatrics: a systematic review
Source: J Glob Health. 2026 Jul 24;16:04186. doi: 10.7189/jogh.16.04186 (PMC13411957; doi:10.7189/jogh.16.04186)
Supplement: Online Supplementary Document [file jogh-16-04186-s001.pdf]

Supplement to: Yao X, Wang Z, Xiong T, Tao M, Hu X. Global trends and career development of human resources for paediatrics: a systematic review. J Glob Health. 2026;16:04186.

Table S1. Search strategy of different database

Database: PubMed

Search number	Query	Sort By	Filters	Search Details	Results	Time	Date
4	#1 AND #2 AND #3			("Paediatrician"[Title/Abstract] OR "Pediatric Workforce"[Title/Abstract] OR "Child Health Workforce"[Title/Abstract]) AND ("Health Personnel"[MeSH Terms] OR "Medical Staff"[MeSH Terms] OR "Health Workforce"[Title/Abstract]) AND ("Internship and Residency"[MeSH Terms] OR "early career"[Title/Abstract] OR "junior doctor"[Title/Abstract] OR "senior consultant"[Title/Abstract])	13	23:53:31	11/19/2025
3	("Internship and Residency"[Mesh] OR "early career"[tiab] OR "junior doctor"[tiab] OR "senior consultant"[tiab])			"Internship and Residency"[MeSH Terms] OR "early career"[Title/Abstract] OR "junior doctor"[Title/Abstract] OR "senior consultant"[Title/Abstract]	71,960	23:10:07	11/19/2025
2	("Health Personnel"[Mesh] OR "Medical Staff"[Mesh] OR "Health Workforce"[tiab])			"Health Personnel"[MeSH Terms] OR "Medical Staff"[MeSH Terms] OR "Health Workforce"[Title/Abstract]	673,782	23:06:05	11/19/2025
1	(Paediatrician[tiab] OR "Pediatric Workforce"[tiab] OR "Child Health Workforce"[tiab])			"Paediatrician"[Title/Abstract] OR "Pediatric Workforce"[Title/Abstract] OR "Child Health Workforce"[Title/Abstract]	2,271	23:01:06	11/19/2025

Database: Embase

Search number	Search Strategy: <1974 to 2025 November 18>	Number
**1**	(Paediatrician or "Pediatric Workforce" or "Child Health Workforce").tw.	(4279)
**2**	exp "Health Personnel"/ or exp "Medical Staff"/ or "Health Workforce".tw.	(2318909)
**3**	exp "Internship and Residency"/ or "early career".tw. or "junior doctor".tw. or "senior consultant".tw.	(415823)
**4**	1 and 2 and 3	(162)

Database: CENTRAL; Date Run: 20/11/2025 13:21:21

ID	Search Hits	Number
#1	(Paediatrician:ti,ab OR "Pediatric Workforce":ti,ab OR "Child Health Workforce":ti,ab)	930

#2	([mh "Health Personnel"] OR [mh "Medical Staff"] OR "Health Workforce":ti,ab)	16749
#3	([mh "Internship and Residency"] OR "early career":ti,ab OR "junior doctor":ti,ab OR "senior consultant":ti,ab)	2190
#4	#1 AND #2 AND #3	2

Database: Scopus

ID	Query: Date 2025 November 20	Number
**1**	((TITLE-ABS(Paediatrician) OR TITLE-ABS("Pediatric Workforce") OR TITLE-ABS("Child Health Workforce")) AND (INDEXTERMS("Health Personnel") OR INDEXTERMS("Medical Staff") OR TITLE-ABS("Health Workforce")) AND (INDEXTERMS("Internship and Residency") OR TITLE-ABS("early career") OR TITLE-ABS("junior doctor") OR TITLE-ABS("senior consultant")))	134
